# Clinical measurements obtained from point-of-care ultrasound images to assess acquisition skills

**DOI:** 10.1186/s13089-019-0119-6

**Published:** 2019-02-22

**Authors:** Brian P. Lucas, Antonietta D’Addio, Clay Block, Harold L. Manning, Brian Remillard, James C. Leiter

**Affiliations:** 1 Medicine Service, White River Junction VA Medical Center, White River Junction, VT USA; 2 Geisel School of Medicine at Dartmouth College, Hanover, NH USA; 3 Department of Medicine, Dartmouth-Hitchcock Medical Center, Lebanon, NH USA

**Keywords:** Clinical competence, Point-of-care systems, Anthropometry, Ultrasonography, Multilevel analysis

## Abstract

**Background:**

Current methods of assessing competence in acquiring point-of-care ultrasound images are inadequate. They rely upon cumbersome rating systems that do not depend on the actual outcome measured and lack evidence of validity. We describe a new method that uses a rigorous statistical model to assess performance of individual trainees based on the actual task, image acquisition. Measurements obtained from the images acquired (the actual desired outcome) are themselves used to validate effective training and competence acquiring ultrasound images. We enrolled a convenience sample of 21 spontaneously breathing adults from a general medicine ward. In random order, two trainees (A and B) and an instructor contemporaneously acquired point-of-care ultrasound images of the inferior vena cava and the right internal jugular vein from the same patients. Blinded diameter measurements from each ultrasound were analyzed quantitatively using a multilevel model. Consistent mean differences between each trainee’s and the instructor’s images were ascribed to systematic acquisition errors, indicative of poor measurement technique and a need for further training. Wider variances were attributed to sporadic errors, indicative of inconsistent application of measurement technique across patients. In addition, the instructor recorded qualitative observations of each trainee’s performance during image acquisition.

**Results:**

For all four diameters, the means and variances of measurements from trainee A’s images differed significantly from the instructor’s, whereas those from trainee B’s images were comparable. Techniques directly observed by the instructor supported these model-derived findings. For example, mean anteroposterior diameters of the internal jugular vein obtained from trainee A’s images were 3.8 mm (90% CI 2.3–5.4) smaller than from the instructor’s; this model-derived finding matched the instructor’s observation that trainee A compressed the vein during acquisition. Instructor summative assessments agreed with model-derived findings, providing internal validation of the descriptive and quantitative assessments of competence acquiring ultrasound images.

**Conclusions:**

Clinical measurements obtained from point-of-care ultrasound images acquired contemporaneously by trainees and an instructor can be used to quantitatively assess the image acquisition competence of specific trainees. This method may obviate resource-intensive qualitative rating systems that are based on ultrasound image quality and direct observation, while also helping instructors guide remediation.

**Electronic supplementary material:**

The online version of this article (10.1186/s13089-019-0119-6) contains supplementary material, which is available to authorized users.

## Background

Point-of-care ultrasound images should reflect patient anatomy and physiology at a given moment in time. Clinical measurements obtained from ultrasound images, however, are known to be highly operator-dependent [1]. The trueness of clinical measurements obtained from ultrasound images should, therefore, be a part of assessments of competence in acquiring them [2]. Reference standards of ‘truth’ are hard to come by, however [3]. Advanced imaging, such as computed tomography or magnetic resonance, is not practical for everyday assessments [4], especially since many point-of-care ultrasound targets are dynamic and require contemporaneous reference standards. Thus, after training programs in point-of-care ultrasound, assessments of trainee competence have not been based on clinical measurements but instead on perceived manual skills or image quality. One potential solution is to have instructors acquire ultrasound images on the same patients at the same time as the trainees under assessment. Clinical measurements obtained from these contemporaneously acquired ultrasound images of an instructor can then serve as reference standards.

## Methods

We describe a method of assessing image acquisition competence for two central veins that are commonly used in bedside assessments of central venous pressure: the inferior vena cava and the right internal jugular vein. Central veins are ideal for studying image acquisition techniques because measurements obtained from ultrasound images of central veins are highly operator-dependent [5]. Our method of performance assessment is derived from statistical modeling of measurements obtained from ultrasound images that were contemporaneously acquired by an instructor and two trainees. We first use this quantitative statistical model to partition measurement errors of ultrasound acquisition for each specific trainee. We then validate these model-derived errors against the instructor’s direct observations of each trainee’s technique acquiring ultrasound images.

### Setting and participants

We conducted this cross-sectional study at a 76-bed, rural, Veterans Affairs hospital during 2 months in 2015. A board-certified internal medicine physician with over 10 years of experience in point-of-care ultrasound [6] served as the instructor. The two trainees were clinical research coordinators who underwent a training program in point-of-care ultrasound. (Details about the training program can be found in Additional file 1: Table S1). The instructor identified a convenience sample of spontaneously breathing adult general medicine inpatients who could comfortably lie supine and had no abdominal or neck tenderness. Given that no identifying information was collected, our internal review board approved participation if patients gave verbal informed consent.

### Ultrasound images

In randomized order and within minutes of each other, each trainee and the instructor acquired ultrasound images on each participant. A full description of the acquisition protocol is presented elsewhere [7]. Briefly, the M-Turbo™ ultrasound (Fujifilm Sonosite, Inc., Bothwell, Washington) was used to acquire 10-s 2-dimensional gray-scale (B-mode) video sequences. A 1–5 MHz phased-array transducer was used to acquire the longitudinal axis of the inferior vena cava from the subcostal window, and a 6–13 MHz linear transducer was used to acquire the transverse axis of the right internal jugular vein from the anterior cervical triangle. Each operator was free to adjust the ultrasound device to optimize the ultrasound image. The image acquisition by all three operators took no more than 10–15 min in any given participant.

All acquired ultrasound images were transferred to a desktop computer without identifying information to ensure that interpretations were not biased by whoever acquired the ultrasound images. The entire 10 s clip of each image was reviewed, and uninterpretable ultrasound images (Additional file 1: Figure S1) were discarded through consensus opinion in group review. All remaining images were randomly arranged and independently interpreted by each trainee and the instructor three times to both limit the influence of outlying measurements and increase the stability of our statistical models. Each vein type was, therefore, measured nine times. The inferior vena cava diameter was measured 3–4 cm from the junction of the right atrium at maximum and minimum excursions, corresponding to end-expiration and end-inspiration, respectively [8]. The mediolateral diameter of the right internal jugular vein was measured when its area was largest, corresponding to end-expiration; from the same still frame, the anteroposterior diameter was measured perpendicular to the midway point of the mediolateral diameter. All diameters were measured from inner edge to inner edge of each vessel.

### Model-derived acquisition-level measurement errors

**Fig. 1:**
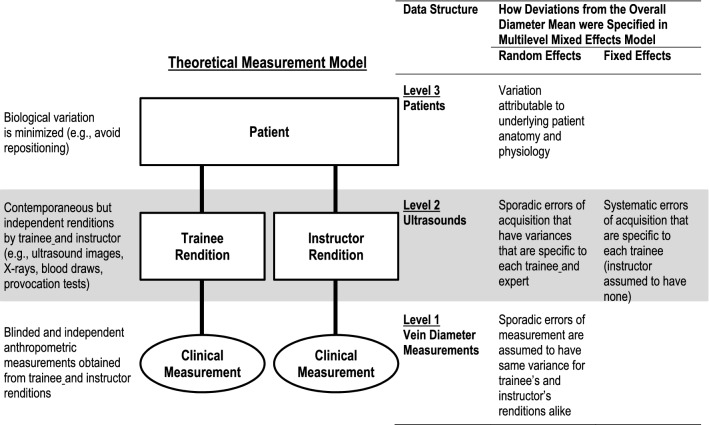
Theoretical measurement model and corresponding specification of multilevel statistical models. *Rectangles* represent groupings (or ‘clusters’), the *ovals* represent vein diameter measurements (the most granular unit of analysis), and *connecting lines* represent nested relationships. In the mixed-effect models, groupings were defined by both random effects and, at level 2 only, fixed effects. There were two fixed effects at level 2, one for each trainee that represented the adjusted differences between diameter means obtained from instructor-acquired ultrasound images. Because instructor-acquired ultrasound images served as the reference standard, the models assume that the instructor made no systematic errors of acquisition. (See text for additional assumptions made for this model.)

We constructed a theoretical measurement model from principles of error analysis [9] and generalizability theory [10] with several standard assumptions (Fig. 1). First, we assumed that measurements reflected not only each patient’s true vein diameters, but also how the ultrasound images were acquired [11]. Second, we ascribed differences between ultrasound images that were acquired on the same patients to acquisition technique rather than substantive biological changes; biological variability within each patient was an improbable source of variation because ultrasound images were acquired contemporaneously and in random order without patient repositioning and while each patient’s volume status was stable [12]. Third, we regarded the instructor’s ultrasound images as the truest representation of each patient’s vein diameters for that moment in time [13]. Fourth, we assumed that the type of difference between trainee- and instructor-acquired ultrasound images (fixed or random) reflected the type of errors being committed by trainees during acquisition: fixed differences reflected systematic errors and random differences reflected sporadic errors [14]. Fifth, we assumed that the techniques used to obtain measurements from images were unaffected by whoever acquired the images, because the person making those measurements was blind to information about who acquired them. Last, we considered diameter measurements on the same ultrasound to be independent of each other because the instructor and trainees worked alone, each obtaining repeated measurements on different days to limit recall of earlier measurements [15]. From this model we devised corresponding statistical models for each of the four vein diameter types. Parameters from the statistical models reflected errors attributable to trainee A’s and trainee B’s images. The primary advantage of this approach is in both quantifying and classifying specific errors of ultrasound acquisition that can then be used to guide the type of remediation best suited to each trainee.

Under an assumption that acquisition technique contributed to measurement error [12], we focused on two comparisons that represented two types of trainee acquisition errors: differences in diameter means reflected one or more systematic errors and differences in the variances of those means reflected one or more sporadic errors. To gauge the magnitude of each trainee’s model-derived acquisition errors, we used thresholds based on the theory of measurement error that have been used for many other types of clinical measurements [9, 16]. We defined each trainee’s *systematic* errors to be acceptable if the magnitude of these errors was less than half a standard deviation of the mean diameter measurements obtained from the instructor’s ultrasound images. Since diameter measurements follow a normal distribution [17], systematic errors of this relative magnitude will misclassify 1 out of 15 vein diameters whenever a reference range is defined as 2 standard deviations above and below a mean [16]. We defined each trainee’s *sporadic* errors to be acceptable when the variance of these errors was less than a quarter of the variance of diameter measurements obtained from the instructor’s ultrasound images across all patients. (This heuristic is based on the simplification that roughly half of the overall variance in measurements is due to variance of measurements within patients.) Sporadic errors with this relative dispersion will add no more than 40% to the overall variability of diameter measurements [16].

We used these thresholds to construct zones of equivalence and noninferiority for systematic and sporadic errors, respectively. Because systematic errors are meaningful in either direction, we used the systematic error thresholds as margins both above and below zero, thereby delineating a zone of equivalence around the adjusted mean obtained from the instructor’s images. For sporadic errors, because variance is inherently ‘one-sided’ (as it is a squared quantity), we used sporadic error thresholds as single margins above zero, delineating the upper bound of zones of noninferiority. We considered model-derived errors to be significant if 90% confidence intervals (CIs) around the point estimates, using two-sided CIs for systematic errors and one-sided CIs for sporadic errors, fell outside zones of equivalence and noninferiority, respectively.

### Instructor-observed acquisition deficiencies

Before the model-derived sources of acquisition errors became available, the instructor observed each trainee’s acquisition technique (Additional file 1: Table S1). The instructor categorized potential deficiencies of each trainee into three domains: placing the ultrasound transducer, confirming visualization, and optimizing ultrasound quality. To avoid influencing each other, the trainees neither observed each other nor the instructor during acquisition of the ultrasound images; nor did they discuss acquisition techniques while awaiting their respective turns to acquire images outside each patient’s room.

### Statistical analysis

Our participant sample size was based on the statistical consideration that at least 12 patients would be needed for unbiased variance estimations [18]; we enrolled more than 12 patients to ensure that we acquired enough evaluable images over a 12-h timeframe. For each diameter measurement, we constructed a separate mixed-effects multilevel model.

The experimental design led to a hierarchical data structure in which the vein diameter measurements themselves where one level nested within the ultrasound image which were in turn nested within the patients (Fig. 1). Patient- and measurement-level random effects were weighted averages (so-called random intercepts), but ultrasound-level random effects were specific to the instructor and the trainee (random coefficients), allowing the magnitude of sporadic errors of acquisition (the variances of ultrasound image random effects) to be compared between the instructor and each trainee. We represented each trainee’s systematic errors of acquisition using fixed effects to allow us to focus specifically on the acquisition performance of the each of the trainees [19]. Intraclass correlation coefficients (ICCs) were derived from models that included fixed effects representing each trainee’s systematic errors of acquisition; this inclusion makes these ICCs so-called ‘consistency ICCs’ [20] because they represent the proportion of total variance that is attributable to patients based on the instructor’s ultrasound images.

For model estimation in our multilevel mixed-effects model, we used a Bayesian estimator because maximum likelihood estimators often fail to converge in three-level models of moderate size. We used the runmlwin [21] command within Stata, version 15.1 (StataCorp, College Station, TX) to run Markov chain Monte Carlo procedures in MLwiN software, version 3.01 (Centre for Multilevel Modeling, Bristol, UK). We used noninformative (or diffuse) priors. Such priors allow the data to drive the results, which will be numerically equivalent to those based on maximum likelihood [22]. Thus, we refer to central ranges of our posterior distributions as ‘confidence intervals,’ even though they are technically Bayesian ‘credibility intervals.’ We report posterior medians rather than means or modes, because medians are less biased for variance components [18].

## Results

**Fig. 2:**
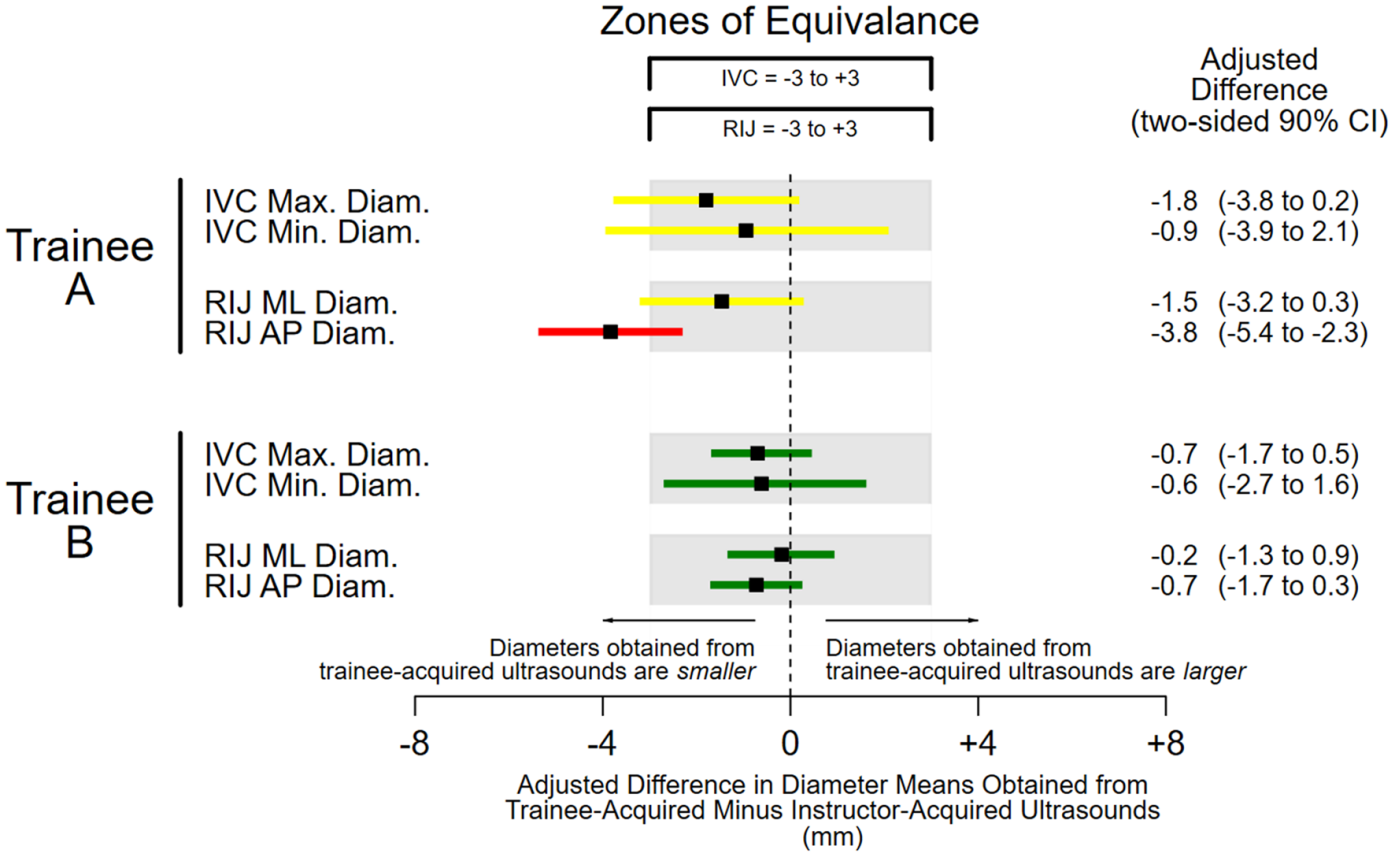
Trainees’ model-derived acquisition-level systematic errors. Shaded areas represent zones of equivalence, which are bounded in each direction by half a standard deviation of mean diameter measurements obtained from the instructor’s ultrasound images (see text for rationale). Color-coded error bars indicate two-sided 90% confidence intervals (CI) that are red if CIs and the point estimates of the mean values are outside equivalence zones; yellow if CIs are outside but point estimates are within the zones; and green if CIs are entirely within the zones. The vertical dashed line represents the adjusted means of diameters obtained from the instructor’s ultrasound images: IVC maximum 16.2 mm (95% CI 13.3–19.1 mm); IVC minimum 10.3 mm (95% CI 6.7–13.9 mm); RIJ mediolateral 14.4 mm (95% CI 11.7–17.0 mm); and RIJ anteroposterior 11.6 mm (95% CI 9.8–13.5 mm). *IVC* inferior vena cava, *RIJ* right internal jugular, *max.* maximum, *min.* minimum, *ML* mediolateral, *AP* anteroposterior, *diam.* diameter

**Fig. 3:**
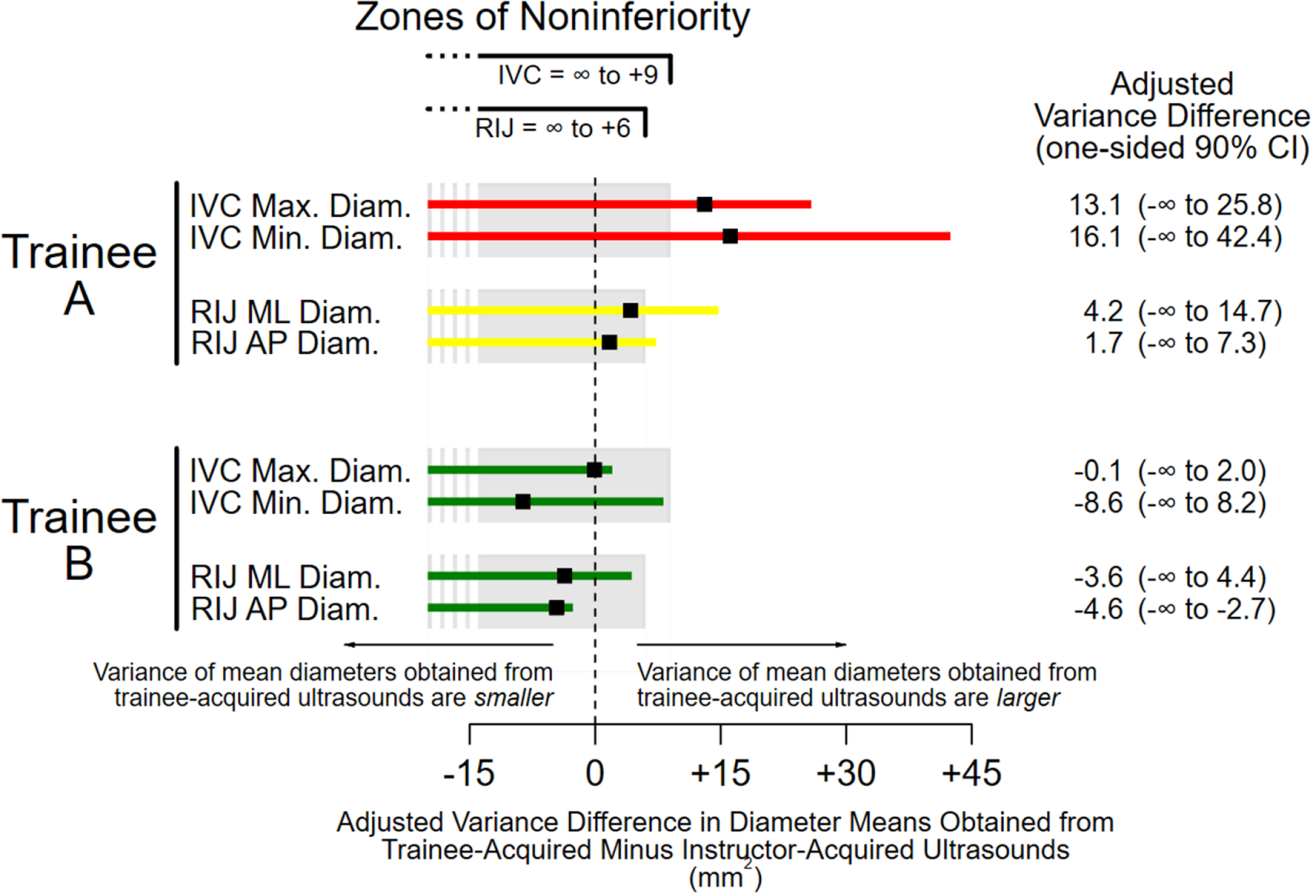
Trainees’ model-derived acquisition-level sporadic errors. Shaded areas represent noninferiority zones, which are bounded only in the direction of larger trainee variance by a quarter of the variance of diameter measurements obtained from the instructor’s ultrasound images (see text for rationale). Color-coded error bars indicate one-sided 90% confidence intervals (CIs) that are red if CIs and the point estimates of variance values are outside noninferiority zones; yellow if CIs are outside but point estimates are within the zones; and green if CIs are entirely within the zones. The vertical dashed line represents the variance in diameter means obtained from instructor-acquired ultrasound images. With the instructor obtaining ultrasound images, intraclass correlation coefficients (the fractions of total variance attributable to patients) for IVC and RIJ veins were 0.88 (95% CI 0.65–0.95) and 0.69 (0.35–0.93) for max. and min. diameters; and 0.83 (0.59–0.99) and 0.63 (0.38–0.83) for ML and AP diameters, respectively. *IVC* inferior vena cava, *RIJ* right internal jugular, *max.* maximum, *min.* minimum, *ML* mediolateral, *AP* anteroposterior, *diam.* diameter

Among 21 participants, the instructor and trainees acquired a total of 39 interpretable inferior vena cava ultrasound images from 16 participants (mean 2.4 examinations per participant, interquartile range [IQR] 2–3) and 48 interpretable internal jugular vein ultrasound images from 18 participants (mean 2.7 examinations per participant, IQR 2–3; Additional file 1: Figure S1). Because the instructor and each trainee obtained diameter measurements from each ultrasound three times, the dataset consisted of 351 inferior vena cava and 432 internal jugular vein ultrasound diameter measurements. The mean inferior vena cava right internal jugular vein diameters (footnote to Fig. 2) suggest that patients had intermediate central venous pressures on average [23, 24]. Nevertheless, the ICCs indicate that 63–88% of variation among vein diameters was attributable to differences between patients (footnote to Fig. 3).

None of the four diameter means obtained from images acquired by trainee A were equivalent to the instructor’s, including the mean of the right internal jugular anteroposterior diameter (− 3.8 mm), which fell below the lower equivalence margin of − 3 mm (Fig. 2). In contrast, all four diameter means obtained from trainee B’s images were equivalent to the instructor’s. A similar pattern was seen with variances. The confidence intervals for all four variances of diameter means obtained from trainee A’s images fell outside the noninferiority zones, making them meaningfully larger than the instructor’s; in fact, even the point estimates fell outside the noninferiority zone for inferior vena cava diameters, (Fig. 3). In contrast, all four variances of diameter means from Trainee B’s images were noninferior to the instructor’s.

Likely proximate effects of the deficiencies of trainees’ acquisition techniques on the measured diameters are listed in Table 1. These qualitative, instructor-observed deficiencies were mapped to the quantitative, model-derived acquisition-level measurement errors. For example, under the assumption that faulty techniques impact ultrasound depictions of vein dimensions, which in turn affect the eventual measurements obtained from those ultrasound images, the deficiencies of trainee A’s techniques likely caused the depiction in the ultrasound images of the anteroposterior diameters of the right internal jugular vein to be smaller than they truly were.

**Table 1:** Mapping of instructor-observed technique deficiencies to model-derived acquisition-level errors

Instructor-observed acquisition technique deficiencies (trainee)^a^	Likely impact on vein dimensions depicted in ultrasound images (diameter types affected)	Model-derived acquisition-level error
Placing transducer		
Off-axis (A)	Smaller due to oblique plane (max, min)	Systematic
Full exhalation not recorded (A)	Not fully enlarged (all)	Systematic
Too much pressure (A)	Compressed (AP)	Systematic
Liver not included for acoustic enhancement (A, B)	Lumen edges indistinct (max, min)	Sporadic
Acoustic ‘rib shadows’ at target (A)	Lumen edges indistinct (max, min)	Sporadic
Not perpendicular to skin of the neck (A)	Lateral lumen edge indistinct (ML)	Sporadic
Not ‘anchored’ during patient movement (A)	Measurement target moving (all)	Sporadic
Confirming visualization		
Incomplete recovery after confirmatory compression (A)	Compressed (AP)	Systematic
Orientation landmarks not included (A, B)	Measurement target unknown (max, min)	Sporadic
Optimizing quality		
Far-field gain too high (A)	Smaller due to far wall artifacts (max, min, AP)	Systematic
Overall gain too high (A, B)	Lumen edges indistinct (all)	Sporadic
Target not centered on screen (B)	Lumen edges indistinct (all)	Sporadic

*Max* maximum diameter of inferior vena cava, *min* minimum maximum diameter of inferior vena cava, *AP* anteroposterior diameter of right internal jugular vein, *ML* mediolateral diameter of right internal jugular vein

^a^Instructor-observed errors related to positioning patients were not included because the instructor and trainees did not reposition patients between acquisitions

## Discussion

We conducted a performance evaluation of ultrasound image acquisition in which the assessment of competence was (a) based on actual performance of the skill being taught, ultrasound image acquisition, and (b) based on clinically meaningful measurements that are derived from the images acquired. Such a direct performance assessment has an advantage over indirect assessment, including direct observations of technique, because the actual results obtained from the activity for which the trainee is being trained are evaluated [2].

Ultrasound measurements of central veins vary greatly between patients. It is worth noting that this variability was incorporated into our statistical model by accounting for the ‘clustering’ of ultrasound images within each patient. This accounting makes the assessment of the acquisition skills of the individual trainees or instructor immune to the large variability of central venous measurements between patients. Moreover, our model-based approach partitioned trainee acquisition errors into systematic errors (consistent deficiencies of performance) and sporadic errors (inconsistent performance techniques from variable technique). Remediation of these two types of errors requires different approaches, and the output of the statistical model can be used, therefore, to focus further training on each trainee’s deficiency (if any).

Thus, we found that clinical measurements obtained from point-of-care ultrasound images reflect image acquisition competence. Specifically, comparisons of four central vein diameters obtained from images acquired contemporaneously by two trainees and an instructor led to quantitative characterizations of each trainee’s skills (Figs. 2 and 3) that were supported by qualitative assessments based on direct observations (Table 1). Acquisition deficiencies observed while trainee A was acquiring images, for example, mapped to both large systematic and large sporadic model-derived measurement errors. Because we were able to quantify the magnitude of trainee A’s acquisition-level measurement errors in the metric used for diameter measurements (mm), and because those measurements follow a normal distribution [17], we can directly estimate how often images obtained by trainee A may be misleading if the underlying deficiencies are not remediated [9]. For example, maximum diameters obtained from trainee A’s inferior vena cava images were systematically smaller than those obtained from the instructor’s images by 1.8 mm (Fig. 2). This means that, in a typical population of spontaneously breathing patients with a mean inferior vena cava maximum diameter of 22 mm and standard deviation of 6 mm [25], 10% of images acquired by trainee A would underestimate central venous pressure. Trainee A would, therefore, misclassify 1 out of 10 patients with truly large inferior vena cava maximum diameters (greater than 20 mm) [23] as having normal-sized diameters (less than or equal to 20 mm). Such misclassifications during management of shock, for example, may lead to inappropriate prescriptions of further volume resuscitation for patients who already have high central venous pressures, possibly worsening their outcomes [26].

Current assessment methods for point-of-care ultrasound acquisition cannot directly quantify the clinical consequences of flawed techniques, because they do not use the actual clinical measurements obtained from the ultrasound images. Instead they use various subjective ratings of acquisition technique [27] and of ultrasound image quality [28]. These subjective ratings have little intrinsic value [2], serving as surrogates for the clinical measurements that ultimately matter most [29]. Summative assessments based on these methods, therefore, will remain subject to claims of arbitrariness, especially without sufficient evidence that such assessments are valid and well correlated with the clinical measurements [30]. We are aware of no subjective rating systems for acquisition of point-of-care ultrasound images that have been compared against contemporaneously acquired reference standards. This gap in validity of subjective assessments is particularly relevant to point-of-care ultrasound because images can appear to be high quality while still misrepresenting the actual in situ anthropometric dimensions. This often occurs, for example, when ultrasound images of cylindrical veins are acquired ‘off-axis,’ generating an oblique view of the vessel that falsely narrows the diameter [5]. In such cases the image may still appear high quality (easily detected edges and good image clarity), belying the flawed acquisition technique and the inaccuracy of the diameter measurement.

Subjective rating systems are also resource-intensive [31]. Raters must be trained and iteratively evaluated for inconsistencies [32]. Although we have not carried out a formal cost comparison, we expect that our method would be less expensive. Instructors simply acquire ultrasound images alongside candidate operators in a dozen participating patients (the sample size that we used in the current study). The only stipulations are that the acquisitions be contemporaneous and independent of each other, which means that (as in our study) ultrasound images be acquired in random order and out of view [12]. Point-of-care ultrasound programs can then focus resources on developing each operator’s skills rather than building an infrastructures of trained raters to perform subjective assessments. Moreover, a similar quantitative assessment of image acquisition skill among instructors could be used to ensure competence and consistency among instructors.

Our study has several limitations. First, we neither collected patient characteristics (such as body habitus) nor incorporated these into our analysis; this potentially limits the generalizability of our findings [33]. Second, we assumed that the instructor’s ultrasound images served as an adequate reference standard, but we did not confirm the accuracy of the measurements obtained from these images [34]. If an instructor and a trainee had a similar deficiency, it would go unnoticed. Third, we assumed that measurements obtained from ultrasound images were independent of who acquired them, because we did not incorporate the interaction term of image-by-interpreter in our multilevel models. However, because we removed all identifying information from the images when they were presented for interpretation in random order, measurement errors introduced by interpretations likely shared a common variance [35]. Fourth, as part of our training program we removed images that were not interpretable in group review (see Additional file 1: Table S1 and Figure S1). Future versions of our method could require trainees to do this independently. Last, we only studied one instructor and two trainees across 21 patients. However, we were not interested in generalizing the performance ‘beyond the sample’ to *other* trainees. The method that we developed is specifically focused on the competence of particular trainees—in our case, trainee A and trainee B—and their systematic and sporadic errors of image acquisition, which can then be remediated; we are not concerned with the population characteristics of all possible trainees. For this reason, we explicitly quantified trainee A’s and trainee B’s systematic and sporadic errors of acquisition by modeling them as ‘fixed effects’ and trainee-specific ‘random coefficients’, respectively [36]. On the other hand, we assumed that our patients were randomly selected from a wider population. Our small sample of patients is realistic, because real-world assessments will always be limited by time and cost constraints. Nonetheless, this small sample led to adequate statistical precision, as our a priori determination predicted.

Future work should aim to address these limitations by expanding our analysis to other representative samples of patients, trainees, and instructors. Strong support for our method would be a demonstration that intentional acquisition deficiencies cause specific model-derived acquisition-level measurement errors. This would then narrow remediation to possible deficiencies that have a causal link to those acquisition-level measurement errors. For example, we observed that measurements from trainee A’s right internal jugular vein were consistently smaller than the instructor’s, suggesting that trainee A was applying too much pressure with the ultrasound transducer (Table 1).

Many clinical decisions rely on clinical measurements, and first steps to improving these decisions require that measurement errors be identified [37]. We believe the quantitative, model-based method of assessment is widely applicable to any operator-dependent skill for which training is required. Like measurements derived from point-of-care ultrasound, many other clinical measurements are susceptible to errors from two distinct sources: the manual technique of acquiring a momentary rendition of a patient at some fixed time, and a cognitive technique that is later applied to interpret those renditions (Fig. 1). Wide-ranging examples include X-rays to measure joint angles, skin scrapings to identify infectious fungi, pressure tracings to estimate attributes of cardiac function, and electrocardiographic stress testing to identify the risk of coronary artery disease. Even routine blood tests are affected not only by the phlebotomists (how long was the tourniquet left in place before blood was drawn?), but also by the laboratory technicians (how long did blood samples sit idle before analysis?) [9]. Each facet of a clinical measurement has the potential to introduce measurement error.

## Conclusions

Our method isolated the errors of the manual technique of acquisition in central vein point-of-care ultrasound. It may also be applied not only to other ultrasound applications beyond central veins but also more widely to assessments of any highly operator-dependent clinical measurements. The primary benefit of using clinical measurements, themselves, in the performance evaluation is that the assessment depends on the relevant performance of the operators, and the contribution of each operator to overall measurement error can be readily partitioned and remediated if deemed substantive.

## Additional file

**Additional file 1: Table S1.** Components of training and how they contributed to competency assessment. **Figure S1.** Flow diagram of inferior vena cava and right internal jugular ultrasounds.

## Abbreviations

CI: confidence interval
IVC: inferior vena cava
RIJ: right internal jugular
max.: maximum
min.: minimum
ML: mediolateral
AP: anteroposterior
diam.: diameter
