# Supplementary material for: Clinical measurements obtained from point-of-care ultrasound images to assess acquisition skills
Source: Ultrasound J. 2019 Feb 22;11:4. doi: 10.1186/s13089-019-0119-6 (PMC6638581; doi:10.1186/s13089-019-0119-6)
Supplement: Supplementary file 1 — Additional file 1: Table S1. Components of training and how they contributed to competency assessment. Figure S1. Flow diagram of inferior vena cava and right internal jugular ultrasounds. [file 13089_2019_119_MOESM1_ESM.docx]

Brian P Lucas, Antonietta D’Addio, Clay Block, Harold Manning, Brian Remillard, and James C Leiter. Clinical measurements obtained from point-of-care ultrasound images to assess acquisition skills

**Table**. Components of training and how they contributed to competency assessment

**Figure**. Flow diagram of inferior vena cava and right internal jugular ultrasounds

**Table.** Components of training and how they contributed to competency assessment

| **Training Component** | **Description (Estimated Time to Complete)** | **Contribution to Competency Assessment** | |
| --- | --- | --- | --- |
|  |  | **Instructor observations** | **Clinical measurements** |
| **Didactic** |  |  |  |
| Reading | Trainees read 50 pages from a text book.^a^ (4 hours) |  |  |
| Viewing | Trainees view 6 screen- and web-cast videos.^b^ (1 hour) |  |  |
| Group Review and “Knobology” | Instructor reviews key textbook images and video segments and orients trainees to the knobs and features of the ultrasound machine and transducers. (2 hours) |  |  |
| **Experiential** |  |  |  |
| Hands-on Training with Instructor | Trainees pair singly with instructor for ultrasound acquisition and interpretation on hospitalized general medicine patients.^c^ (15 hours^d^) | **✓** |  |
| Independent Acquisition | In random order and within minutes of each other, trainees and instructor independently attempt to acquire ultrasound images on 21 hospitalized general medicine patients.^e^ (12 hours^d,f^) |  | **✓**  (images acquired) |
| Group Review of Ultrasounds for Interpretability | Working as a group and blind to acquirer, trainees and instructor review ultrasound images, excluding those not interpretable. (3 hours) |  |  |
|  | After unblinding to acquirer, group discusses flaws in techniques and how to avoid them. (1 hour) | **✓** |  |
| Independent Interpretation | Trainees and instructor independently interpret the same set of ultrasound images (39 inferior vena cava and 48 right internal jugular) 3 times. (12 hours) |  | **✓**  (measurements obtained) |
| Group Review of Ultrasounds with Highest Variability | After unblinding to interpreter, ultrasound images with interpretations in the upper quarter of variability^g^ are reviewed together by trainees (3 hours) |  |  |
|  | Group discusses flaws in techniques and how to avoid them. (1 hour) | **✓** |  |
| **Total Time** | **54 hours** |  |  |

**Table (continued)**

a Soni NJ, Arntfield R. Point of care ultrasound. Philadelphia, PA: Elsevier Saunders; 2015: 3–45, 135–141.)

b 1) Lewiss RE. Ultrasound Physics and Machinery. Society of Academic Emergency Medicine Lecture Series. https://vimeo.com/94786374. Accessed May 19, 2017. 2) Fischer JI. Inferior Vena Cava Ultrasound. Society of Academic Emergency Medicine Lecture. https://vimeo.com/59095991. Accessed May 19, 2017. 3) Ultrasound Critical Care. How to obtain: Inferior Vena Cava Ultrasound View—Training and Techniques. https://www.youtube.com/watch?v=McUUFvnFuJU. Accessed May 19, 2017. 4) SonoSite. 3D How To: Inferior Vena Cava Ultrasound Exam. https://www.youtube.com/watch?v=ci9W4MvyMHI. Accessed May 19, 2017. 5) Greater New York Hospital Association Project. IVC Ultrasound for Fluid Responsiveness in Spontaneously Breathing Patients. https://www.youtube.com/watch?v=ndcJ4DjmWVY. Accessed May 19, 2017. 6) Croft P. Massachusetts General Hospital Emergency Department. Aorta and Inferior Vena Cava. https://www.youtube.com/watch?v=_9nxXp5Kyug. Accessed May 19, 2017.

c Hands-on training for trainees A and B occurred on 17 and 21 patients, respectively, during 5 sessions each lasting 3 hours. The first 4 hands-on sessions focused on acquisition and occurred at patients’ bedsides. To provide each trainee with a goal of 20 training acquisitions, approximately 2 general medicine inpatients were enrolled per hour. The instructor directed trainees step-by-step through patient positioning, transducer handling, and image optimization.

d Included time for verbal consent process.

e The hospitalized general medicine patients enrolled during independent acquisition were not the same patients enrolled during hands-on training with the instructor.

f Included time waiting for instructor and other trainee to acquire ultrasound images on the same patients.

g Variability in interpretation of an ultrasound image was defined as the standard deviation of the mean of the 3 measurement made by each trainee and the instructor. Ultrasound images were ordered by the magnitude of their standard deviations and the upper quarter of standard deviations were selected for review.

**Figure.** Flow diagram of inferior vena cava and right internal jugular ultrasound images

^a^Trainees had completed a didactic introduction and 15 hours of hands-on training prior to acquisition of ultrasound images. ^b^Each of the 21 enrolled patients was assigned a letter from A to U. ^c^The inferior vena cava could not be visualized for patients L, M, and S. ^d^The right internal jugular vein was could not be visualized for patients L, M, and T. Fifteen inferior vena cava ultrasounds were excluded among 9 unique patients because of overall poor quality of the ultrasound images acquired by trainee A (patients D, H, and Q), trainee B (patients D and F), and instructor (patients Q and U); of inadequate visualization of the measurement site for ultrasound images acquired by trainee A (patient U), trainee B (patients A, P, and U), and instructor (patients A and T); and the aorta but not the inferior vena cava was recorded on ultrasounds acquired by trainee B (patients J and Q).

**Figure**

**Trainee A^a^**

**Instructor**

**Trainee B^a^**

**Acquire Ultrasound Images on 21 Hospitalized General Medicine Patients ^b^**

In random order and within minutes of each other trainees and instructor attempt to independently acquire inferior vena cava (IVC) and right internal jugular (RIJ) vein ultrasound images

**18 IVC^c^**

**18 RIJ^d^**

**18 IVC^c^**

**18 RIJ^d^**

**18 IVC^c^**

**18 RIJ^d^**

**Review Quality of 54 IVC and 54 RIJ Ultrasound Images**

Blinded to who acquired the ultrasound images and working as a group, trainees and instructor ensure interpretability of each image. Through group discussion instructor gathers potential errors of acquisition.

**4 IVC excluded ^d^**

**(patients A, Q, T, U)**

**14 interpretable IVC**

**2 RIJ excluded ^d^**

**(patients C, D)**

**16 interpretable RIJ**

**4 IVC excluded ^d^**

**(patients D, H, Q, U)**

**14 interpretable IVC**

**4 RIJ excluded ^d^**

**(patients A, E, H, S)**

**14 interpretable RIJ**

**11 interpretable IVC**

**7 IVC excluded ^d^**

**(patients A, D, F, J, P, Q, U)**

**0 RIJ excluded ^d^**

**18 interpretable RIJ**

**Measure Diameters of 39 IVC and 48 RIJ Ultrasound Images**

Blinded to who acquired the ultrasound images, trainees and instructor independently measure 2 diameters for each interpretable image 3 separate times. Thus 9 measurements per ultrasound are generated for each diameter type.

**351 measurements of IVC maximum and minimum diameters**

**432 measurements of RIJ anteroposterior and mediolateral diameters**

**Model 351 IVC and 432 RIJ Diameter Measurements**

Separate 3-level mixed-effects models were built for each of 4 different diameter measurements (see *Figure 1*).
